# Supplementary material for: Excessive serine from the bone marrow microenvironment impairs megakaryopoiesis and thrombopoiesis in Multiple Myeloma
Source: Nat Commun. 2023 Apr 13;14:2093. doi: 10.1038/s41467-023-37699-z (PMC10102122; doi:10.1038/s41467-023-37699-z)
Supplement: Supplementary file 1 — Supplementary Information [file 41467_2023_37699_MOESM1_ESM.pdf]

Supplementary Information

Excessive serine from the bone marrow microenvironment impairs megakaryopoiesis and thrombopoiesis in Multiple Myeloma

Chunmei Kuang#, Meijuan Xia#, Gang An, CuiCui Liu, Cong Hu, Jingyu Zhang, Zhenhao Liu, Bin Meng, Pei Su, Jiliang Xia, Jiaojiao Guo, Yinghong Zhu, Xing Liu, Xuan Wu, Yi Shen, Xiangling Feng, Yanjuan He, Jian Li, Lugui Qiu, Jiaxi Zhou*, Wen Zhou*

Supplementary Figures

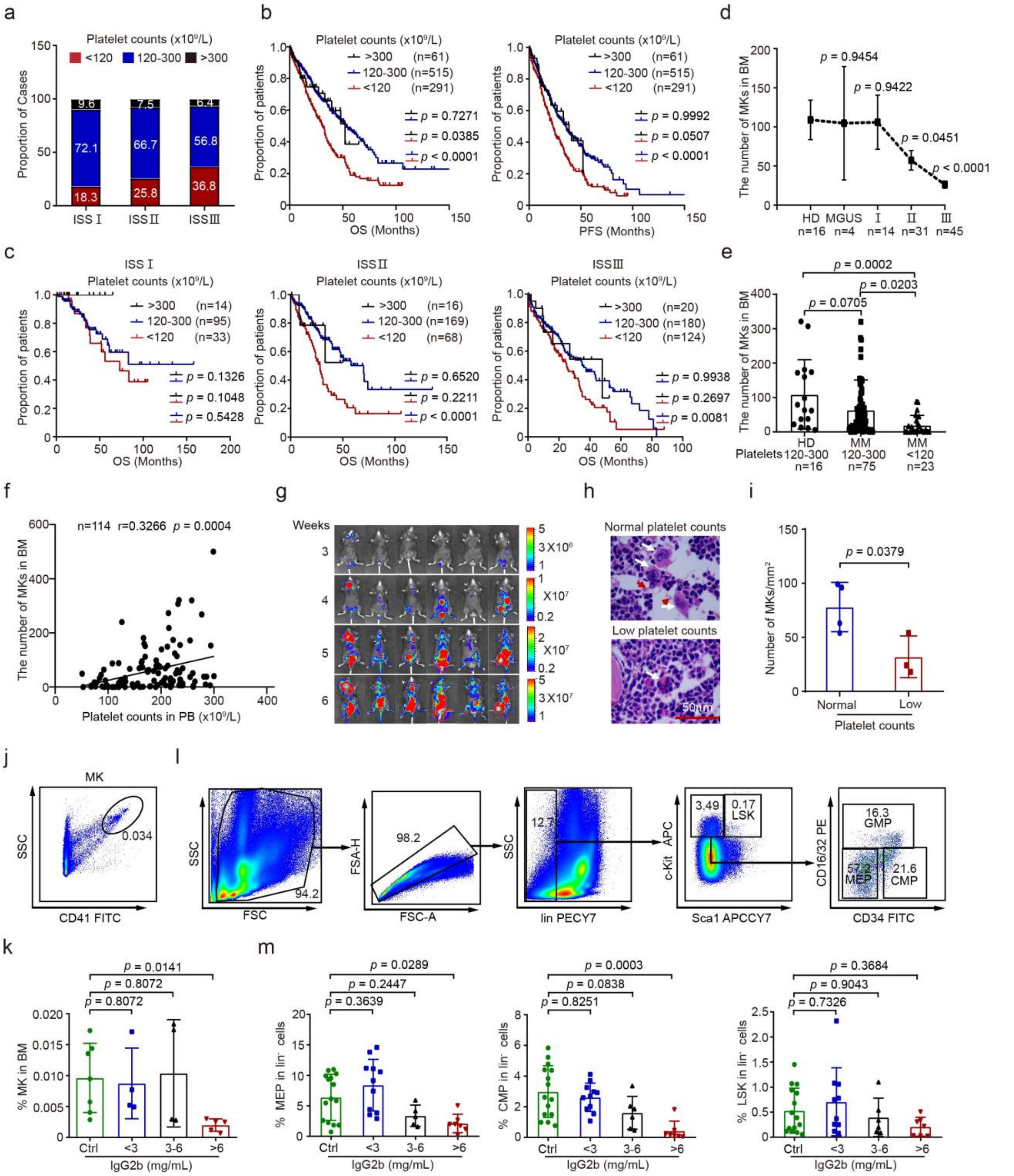

Supplementary Figure 1 Thrombocytopenia is associated with poor prognosis in MM. a The proportion of NDMM patients with normal platelet counts (120-300×10^9/L), low platelet counts (≤120×10^9/L) and high platelet counts (>300×10^9/L) at different ISS stages (nMM (ISS I) = 142, nMM (ISS II) = 253, nMM (ISS III) = 324). **b** Kaplan-Meier analysis of overall survival (OS, left) and progression-

free survival (PFS, right) in NDMM patients with normal platelet counts (120-300×10^9/L, n = 515), high platelet counts (>300×10^9/L, n = 61) and low platelet counts (≤120×10^9/L, n = 291). **c** Kaplan-Meier analysis of OS in MM patients with normal platelet counts (120-300×10^9/L, nMM (ISS I) = 95, nMM (ISS II) = 169, nMM (ISS III) = 180), high platelet counts (>300×10^9/L, nMM (ISS I) = 14, nMM (ISS II) = 16, nMM (ISS III) = 20), and low platelet counts (≤120×10^9/L, nMM (ISS I) = 33, nMM (ISS II) = 68, nMM (ISS III) = 124) at different ISS stages. **d** The number of MKs in BM aspirate smears derived from healthy donors (HD), MGUS (monoclonal gammopathy of undetermined significance) and NDMM patients with different ISS stages (mean ± SD, nHD = 16, nMGUS=4, nMM (ISS I) = 14, nMM (ISS II) = 31; nMM (ISS III) = 45). **e** The number of MKs in BM aspirate smears derived from HD and MM patients with normal platelet counts and low platelet counts (mean ± SD, nHD = 16, nMM (normal) = 75, nMM (low) = 23). **f** The assessment of the correlation between platelet counts and numbers of MKs in BM (n = 114, two-sided Pearson test). **g** Tumor-associated luminescence intensity in live 5TGM1 mice at week 3, 4, 5 and 6 (n = 6). **h** Representative images of MKs in the BM from 5TGM1 mice with normal platelet counts or low platelet counts by using Hematoxylin staining. **i** Statistical analysis of the number of MKs per mm^2 in the BM of 5TGM1 mice with normal platelet counts (n = 4) or low platelet counts (n = 3). **j** Representative plot for MKs was detected in 5TGM1 mice. CD41 positive cells with high SSC are MKs. **k** Flow cytometry analysis of the proportion of MKs in BM from the control mice and 5TGM1 mice with different tumor burden (mean ± SD, n = 7 in ctrl group, n = 4 in group with IgG2b less than 3mg/mL, n = 4 in group with IgG2b range from 3 to 6mg/mL, n = 5 in group with IgG2b more than 6mg/mL). Results represent means ± SD. **l** Gating strategy for LSK (Lin^-/$Sca\text{-}1^+$/$c\text{-}Kit^+$), CMPs (Lin^-/$Sca\text{-}1^-$/$c\text{-}Kit^+$/$CD34^+$/$CD16/32^-$) and MEPs (Lin^-/$Sca\text{-}1^-$/c-Kit^+/$CD34^-$/ $CD16/32^-$). **m** Flow cytometry analysis of the proportion of LSKs, CMPs, and MEPs in the BM from the control mice and 5TGM1 mice with different tumor burden (mean ± SD, n = 15 in ctrl group, n = 11 in group with IgG2b less than 3 mg/mL, n = 6 in group with IgG2b range from 3 to 6 mg/mL, n = 7 in group with IgG2b more than 6 mg/mL). Unpaired two-sided t-test were used in **d, e, i, k, m;** Two-sided log-rank (Mantel-Cox) test were used in **b, c.** Source data are provided as a Source Data file.

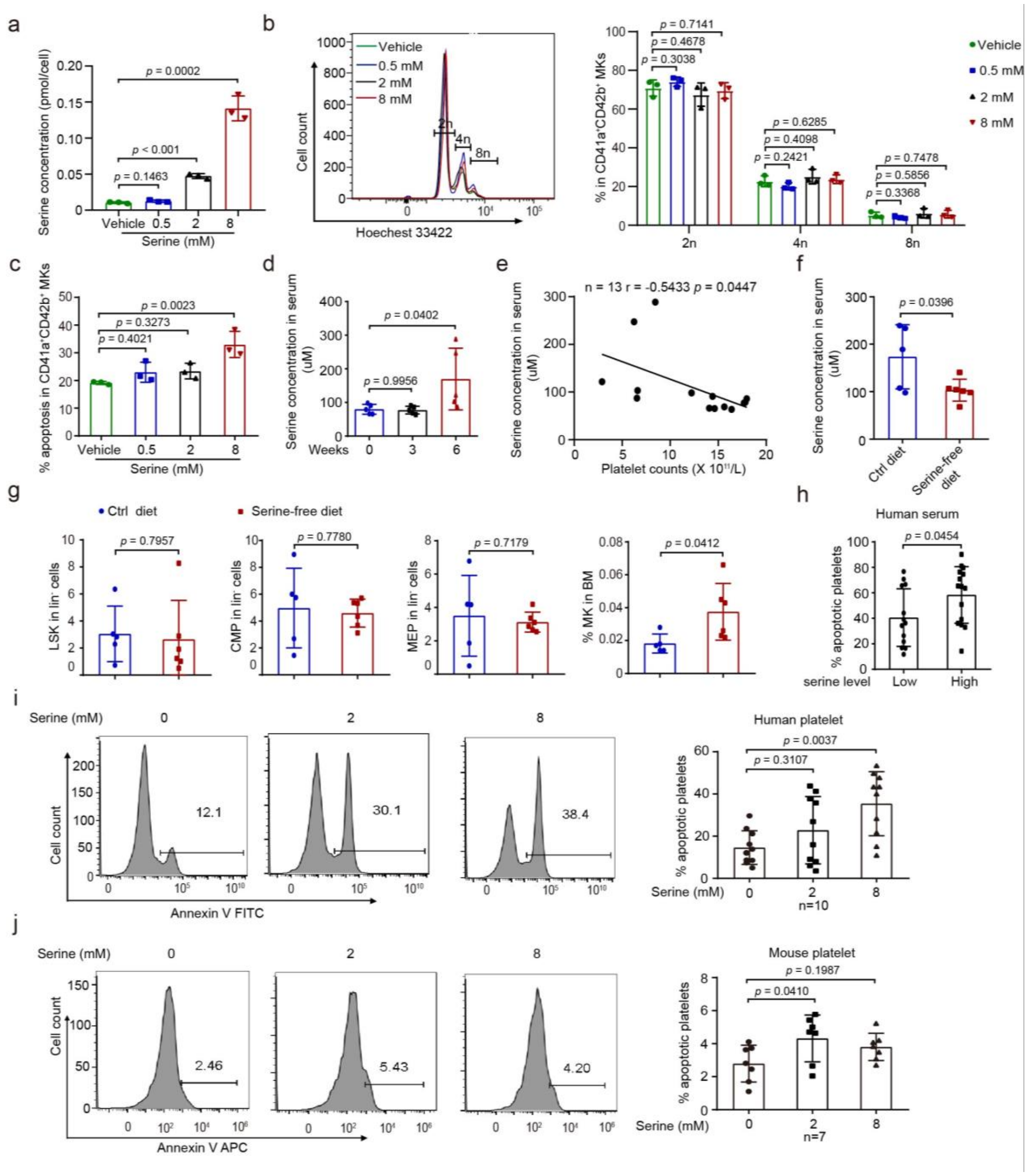

Supplementary Figure 2 High serine is linked to thrombocytopenia and induces platelet apoptosis. a Serine levels in differentiated cells treated with various concentrations of serine, as determined with high-performance liquid chromatography (HPLC) (mean ± SD, n = 3 independent experiments). **b** The ploidy of differentiated cells at day 12 treated with vehicle or serine (mean ± SD, n = 3 independent experiments). **c** The apoptosis rate of CD41a^{+}CD42b^{+} cells at day 12 of treatment with vehicle or serine (mean ± SD, n = 3 independent experiments). **d** The level of serine in the serum of 5TGM1 mice at week 0, 3, 6 was detected by using HPLC (mean ± SD, n = 5). **e** The correlation between platelet counts and serum serine level in 5TGM1 mice (n = 13). **f** The serum serine level in 5TGM1 mice fed with

the control diet or serine-free diet, as detected with HPLC (mean ± SD, n = 5). **g** The proportion of LSK, CMP, MEP and MK in 5TGM1 mice fed with the control diet or serine-free diet (mean ± SD, n = 5). **h** Statistical analysis of the percentage of apoptotic platelets in HD-derived platelets incubated with serum from MM patients with low or high serine levels (mean ± SD, nMM_{low} = 13, nMM_{high} = 15). **i, j** Gating strategy for Annexin V^+ apoptotic platelets and statistical analysis of the percentage of apoptotic platelets in HD-derived platelets and control mouse-derived platelets incubated with vehicle, 2mM and 8mM serine. Results represent means ± SD. Unpaired two-sided t-test were used in **f, g, h;** One-way ANOVA followed by Dunnett's multiple comparison test were used for **a, b, c, d, i, j**; Two-sided Pearson test was used in **e**. Source data are provided as a Source Data file.

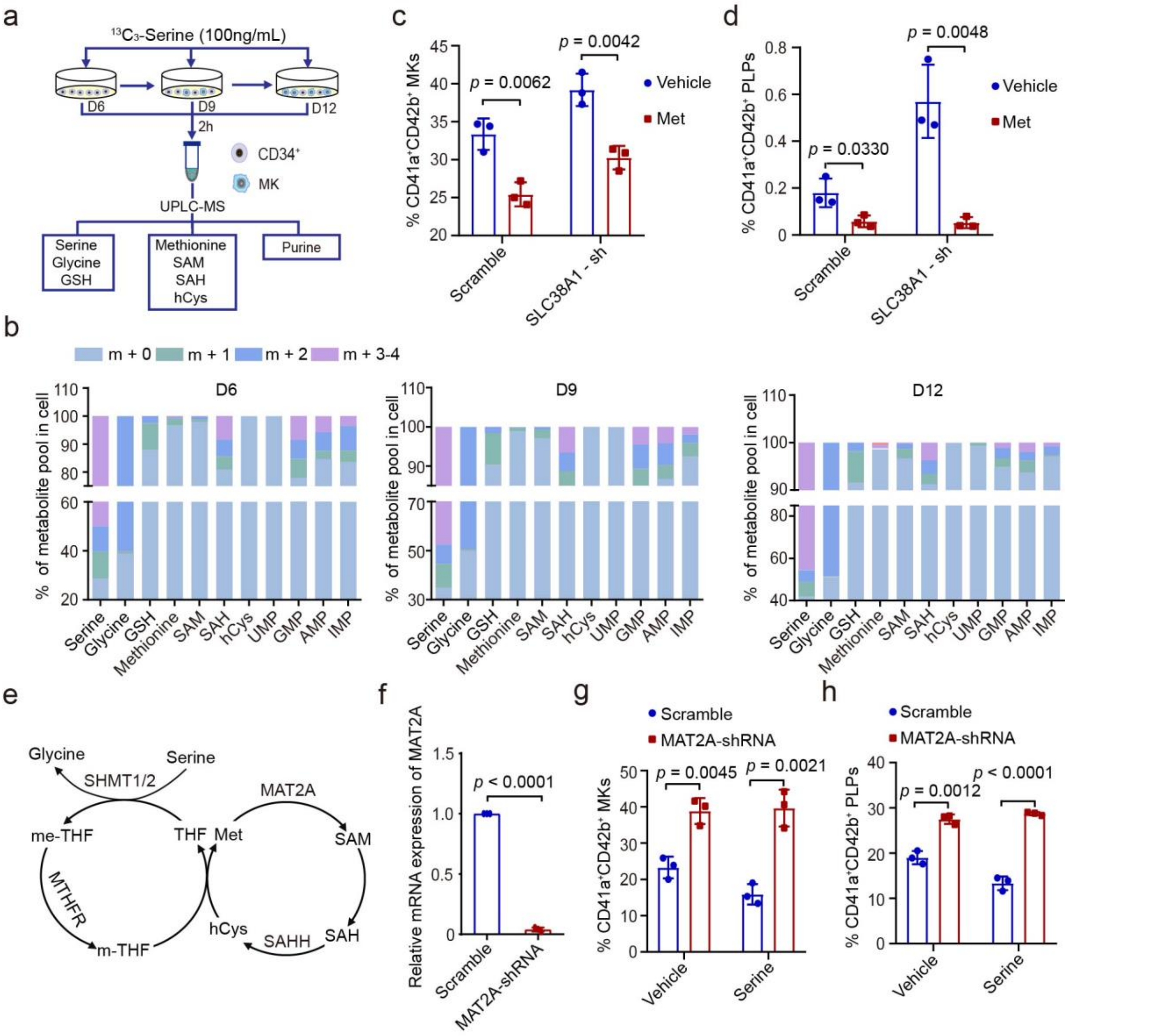

Supplementary Figure 3 Serine entry into one-carbon metabolism in cells undergoing megakaryocytic differentiation. a

Schematics of the serine metabolic flux experiments. **b** The proportions of 13C-labeled metabolites in $CD34^+$ cells undergoing MK differentiation (at day 6, 9 and 12) after the exposure to $^{13}C_3$-serine for 2 h. **c** Generation of $CD41a^+CD42b^+$ cells at day 12 from $CD34^+$ cells infected with lentivirus containing Scramble and SLC38A1-shRNA in the presence of vehicle or methionine (Met) (mean ± SD, n = 3 independent experiments). **d** Generation of $CD41a^+CD42b^+$ PLP at day 12 from $CD34^+$ cells infected with lentivirus containing Scramble and SLC38A1-shRNA in the presence of vehicle or Met (mean ± SD, n = 3 independent experiments). **e** Overview of one-carbon metabolism around the folate cycle and methionine cycle. **f** Assessment of mRNA level of *MAT2A* in differentiated cells treated with vehicle or serine at day 12 by using RT-qPCR (mean ± SD, n = 3 independent experiments). **g** Flow cytometry analysis of the percentage of $CD41a^+CD42b^+$ MKs from $CD34^+$ cells infected with Scramble or MAT2A-shRNA virus in the presence vehicle or serine (mean ± SD, n = 3 independent experiments). **h** Generation of $CD41a^+CD42b^+$ PLPs at day 12 from $CD34^+$ cells infected with Scramble or MAT2A-shRNA virus in the presence vehicle or serine (mean ± SD, n = 3 independent experiments). Unpaired two-sided t-test were used in **c**, **d, f, g, h.** Source data are provided as a Source Data file.

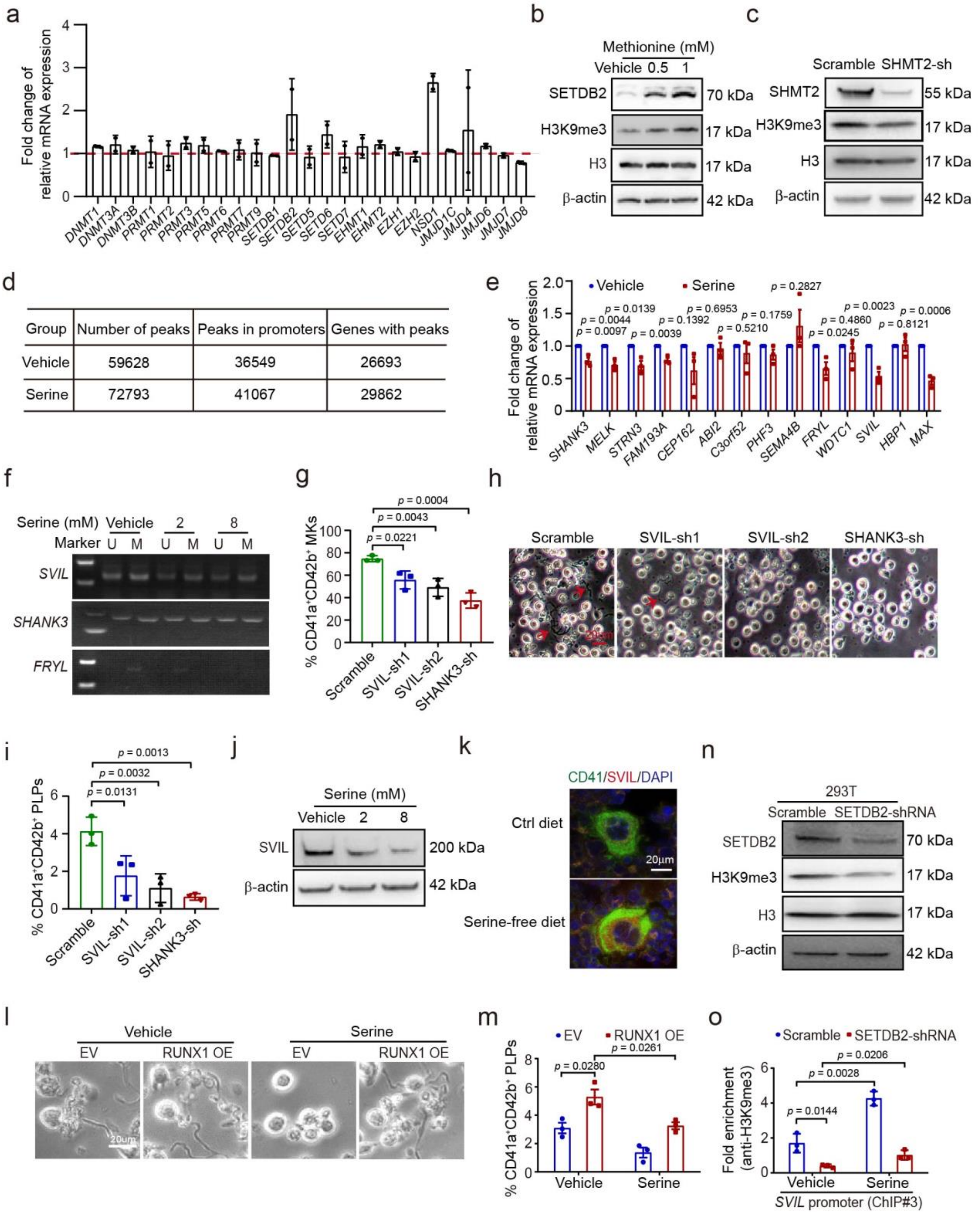

Group	Number of peaks	Peaks in promoters	Genes with peaks
Vehicle	59628	36549	26693
Serine	72793	41067	29862

Supplementary Figure 4 Serine downregulates Supervillin via S-adenosyl-methionine-mediated tri-methylation of H3K9.

a Relative mRNA levels of methyltransferase were detected in differentiated cells treated with vehicle or 8mM serine at day 12 by using RNA-seq (mean ± SD, $n = 2$ independent experiments). **b** Protein levels of SETDB2 and H3K9me3 were detected with immunoblotting in differentiated cells treated with vehicle or methionine at day 12. **c** Protein levels of SHMT2 and H3K9me3 were detected with

immunoblotting in differentiated cells infected with Scramble or SHMT2-shRNA at day 12. **d** The distribution of peaks in differentiated cells treated with vehicle or 8mM serine. **e** Relative mRNA levels of predicted downregulated genes were detected in differentiated cells treated with vehicle and serine by using RT-qPCR (mean ± SD, $n = 3$ independent experiments). **f** The methylation status of the CpG islands in *SVIL*, *FRYL* and *SHANK3* promoters in differentiated cells treated with vehicle or serine, detected using Methylation-Specific PCR (MS-PCR). **g** Generation of CD41a$^+$CD42b$^+$ cells at day 12 from CD34$^+$ cells infected with lentivirus containing scramble and SVIL-shRNA or SHANK3-shRNA (mean ± SD, $n = 3$ independent experiments). **h, i** Phase-contrast images of proplatelet formation and generation of CD41a$^+$CD42b$^+$ PLPs at day 12 from CD34$^+$ cells infected with lentivirus containing scramble, SVIL-shRNA or SHANK3-shRNA (mean ± SD, $n = 3$ independent experiments). The scale bar is 20 μm. **j** Protein levels of SVIL were detected with immunoblotting in differentiated cells treated with vehicle or serine at day 12 of differentiation. **k** Protein levels of SVIL were detected with immunofluorescence in CD41$^+$ cells of 5TGM1 mice fed with ctrl diet or serine-free diet. The scale bar is 20 μm. **l** Phase-contrast images of proplatelet formation at day 12 from CD34$^+$ cells infected with lentivirus with EV or RUNX1 in the presence or the absence of serine. The scale bar is 20 μm. **m** CD41a$^+$CD42b$^+$ PLPs at day 12 from CD34$^+$ cells infected with lentivirus with EV or RUNX1 in the presence or the absence of serine (mean ± SD, $n = 3$ independent experiments). **n** Protein levels of SETDB2 and H3K9me3 were detected with immunoblotting in 293T cells infected with lentivirus containing scramble or SETDB2-shRNA. **o** The binding of H3K9me3 to the promoters of the *SVIL* in 293T cells infected with lentivirus with scramble or SETDB2-shRNA in the presence or the absence of serine, as revealed with ChIP-qPCR (mean ± SD, $n = 3$ independent experiments). Each experiment was repeated independently for 3 times in **b, c, f, j, k, n**. Unpaired two-sided *t*-test were used in **e**, **m, o;** One-way ANOVA followed by Dunnett's multiple comparison test were used for **g**, **i**. Source data are provided as a Source Data file.

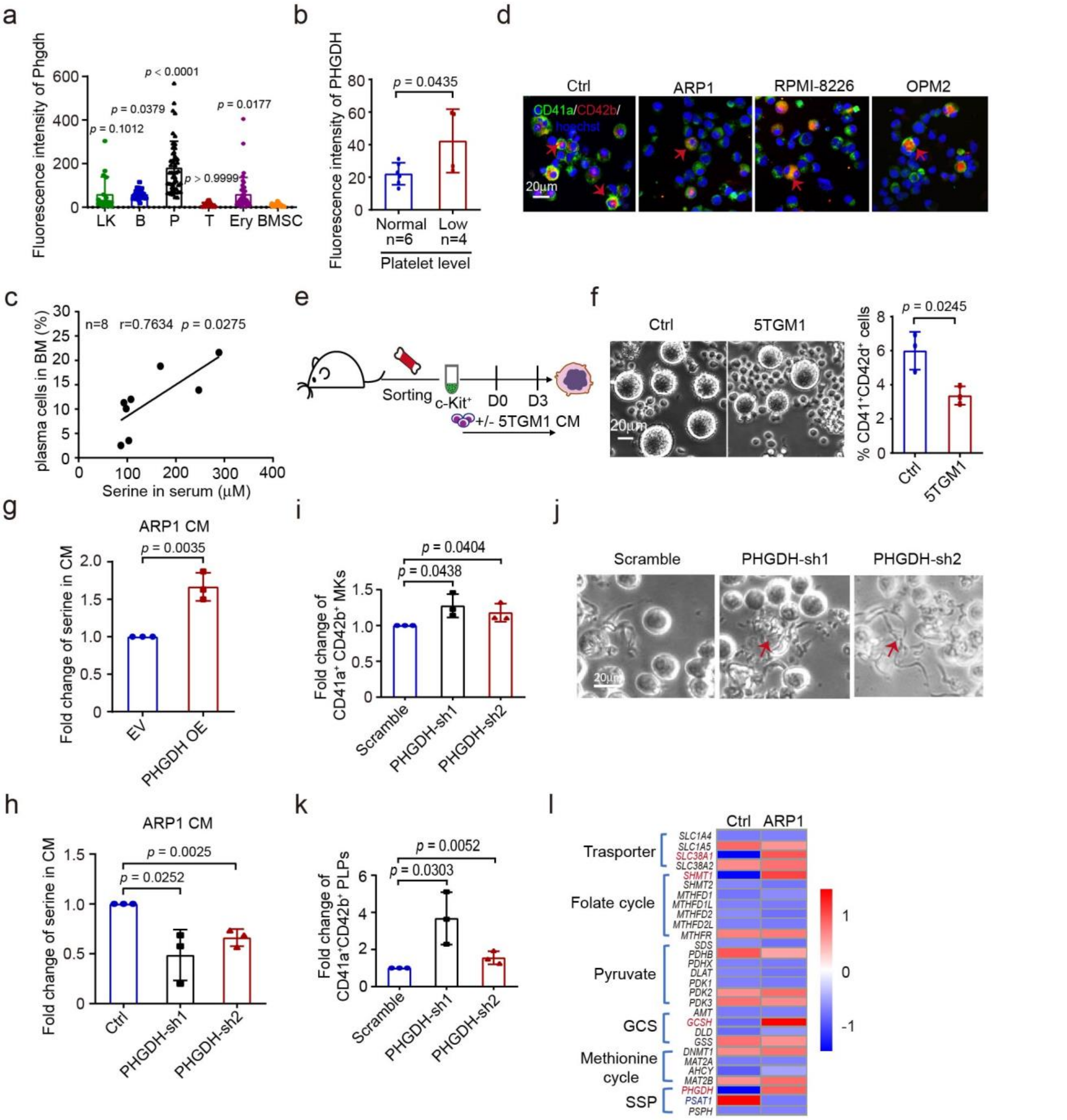

Supplementary Figure 5 Serine accumulated in the BM microenvironment is released from myeloma cell. a Fluorescence intensity of Phgdh in LK, B, P, T, Ery and BMSC in the BM of 5TGM1 mice (n = 18 images in LK, n = 34 images in B cells, n = 49 images in plasma cells, n = 21 images in T cells, n = 44 images in erythrocytes, n = 30 images in BMSCs). BMSCs were used as control cells. Results represent means ± SD, Significance was analyzed with One-way ANOVA followed by Dunnett's multiple comparison test. **b** The fluorescence intensities of PHGDH in $CD138^+$ cells derived from MM patients with normal platelet counts and low platelet counts. **c** The correlation between the percentage of $CD138^+$ plasma cells in the BM and serum serine level of 5TGM1 mice (n = 8). **d**

Representative images of immunofluorescence stained with CD41a and CD42b at day 12 in differentiated cells treated with CM of MM cells. The scale bar is 20 μm. Experiment was repeated independently for 3 times. **e** Schematics of MK differentiation of c-Kit$^+$ cells treated with the CM derived from 5TGM1 cells. **f** Phase-contrast images of MK cells (left) and generation of CD41a$^+$CD42b$^+$ cells (right) from c-Kit$^+$ cells treated with control medium or 5TGM1 CM (mean ± SD, $n = 3$ independent experiments). The scale bar is 20 μm. **g** The level of serine in CM from ARP1-EV cells and ARP1-PHGDH-OE cells, as determined with HPLC ($n = 3$ independent experiments). **h** The level of serine in CM from ARP1-Scramble cells and ARP1-PHGDH-shRNA cells, as determined with HPLC (mean ± SD, $n = 3$ independent experiments). **i** Flow cytometry analysis of the percentage of CD41a$^+$CD42b$^+$ MKs from CD34$^+$ cells treated with ARP1-PHGDH-sh1 CM and ARP1-PHGDH-sh2 CM (mean ± SD, $n = 3$ independent experiments). **j, k** Representative images of proplatelet formation and generation of CD41a$^+$CD42b$^+$ PLPs at day 12 from CD34$^+$ cells treated with ARP1-PHGDH-sh1CM and ARP1-PHGDH-sh2 CM (mean ± SD, $n = 3$ independent experiments). The scale bar is 20 μm. **l** The expression profile of serine metabolism-related genes in differentiated cells at day 12 cultured with the control medium or ARP1 CM. Unpaired two-sided *t*-test were used in **b**, **f, g, h, i, k.** Two-sided Pearson test was used in **c**. Source data are provided as a Source Data file.

Supplementary Tables

Supplementary Table 1. Multivariate Cox logistic regression analysis of OS and PFS and platelet counts

Variable	OS		PFS	
	HR (95%CI)	*p*-value	HR (95%CI)	*p*-value
Gender (M vs. F)	0.882 (0.626-1.241)	0.471	1.194 (0.873-1.633)	0.267
Age (>50 vs. ≤50)	1.023 (1.006-1.040)	0.006**	1.012 (0.997-1.027)	0.113
1q21 gain (0 vs.1)	1.416 (1.006-1.994)	0.046*	1.568 (1.156-2.127)	0.004**
TP53 deletion (0 vs.1)	1.87 (1.198-2.920)	0.006**	2.29 (1.54-3.405)	0.001**
Platelets (Normal vs. Low)	0.597 (0.406-0.878)	0.009**	0.605 (0.427-0.858)	0.005**

Abbreviations: 1q21 gain: FISH 1q21 gain in ≥10% of cells; TP53 deletion: FISH TP53 deletion in ≥10% of cells; OS: overall survival; PFS: progression-free survival; all MM patients were newly diagnosed. $*p < 0.05$, $**p < 0.01$, $***p < 0.001$. Significance was calculated with Cox Regression analysis.

Supplementary Table 2. Correlation between platelet counts, MK counts and clinical characteristics

Patients' Characteristics	Healthy Donors (n=16) (n/N×100%)	Normal platelet counts (n=75) (n/N×100%)	Low platelet counts (n=23) (n/N×100%)	*p*-value
Male (%)	10/16 (62.5)	41/75 (64)	10/23 (43.5)	0.876[a]
Age (y)	40	56.8	59	0.359[b]
ISS stage				0.042[a]*
I		11/63 (16.1)	1/21 (5)	
II		25/63 (38.7)	4/21 (20)	
III		27/63 (45.2)	16/21 (75)	
Plasma cells in BM (%)		21.7	39.3	0.079[b]
Median Hb (g/L)		108.8	82.3	0.000[b]***
Median MKs	109	64	20	0.02[b] *

Abbreviations: ISS: International Staging System; y, years. Hb： hemoglobin; all MM patients were newly diagnosed. $*p < 0.05$, $**p < 0.01$. [a], Two-tailed Pearson Chi-Square test; [b], Unpaired two-sided Student's *t*-test.

Supplementary Table 3. Correlation between platelet counts, serine and clinical characteristics

Patients' Characteristics	Low serine in BM		High serine in BM		p-value
	Normal platelet (n=68, %)	Low platelet (n=22, %)	Normal platelet (n=15, %)	Low platelet (n=9, %)	
Age (y)	59	57	58	51	0.257[a]
Sex					0.803[b]
Male	35/68 (51.5)	12/22 (54.5)	7/15 (46.7)	6/9 (66.7)	
Female	33/68 (48.5)	10/22 (45.5)	8/15 (53.3)	3/9 (33.3)	
ISS stage					0.048[b]*
I	14/66 (21.2)	3/22 (13.6)	5/15 (33.3)	0/9 (0)	
II	16/66 (24.2)	8/22 (36.4)	7/15 (46.7)	1/9 (11.1)	
III	36/66 (54.6)	11/22 (50)	3/15 (20)	8/9 (88.9)	
DS stage					0.838[b]
I	4/66 (6.1)	0/22 (0)	1/15 (6.7)	0/9 (0)	
II	4/66 (6.1)	1/22 (4.5)	1/15 (6.7)	0/9 (0)	
III	58/66 (87.8)	21/22 (95.5)	13/15 (86.6)	9/9 (100)	
Plasma cells in BM (%)	44.75	56.86	41.7	56.67	0.429[a]
Median Hb (g/L)	98.3	78	107.6	75.3	0.000[a]***
Renal dysfuction					0.304[b]
No	48/66 (72.7)	18/22 (81.8)	13/15 (86.7)	5/9 (55.6)	
Yes	18/66 (27.3)	4/22 (18.2)	2/15 (13.3)	4/9 (44.4)	
Median LDH (U/L)	176.1	249.9	166.8	231.1	0.317[a]
Median Calcium (mmol/L)	2.41	2.25	2.31	2.09	0.06[a]
1q21 gain					0.736[b]
No	27/66 (40.9)	10/22 (45.5)	8/15 (53.3)	5/9 (55.6)	
Yes	39/66 (59.1)	12/22 (54.5)	7/15 (46.7)	4/9 (44.4)	
*TP*53 deletion					0.504[b]
No	57/66 (86.4)	18/21 (85.7)	15/15 (100)	7/8 (87.5)	
Yes	9/66 (13.6)	3/21 (14.3)	0/15 (0)	1/8 (12.5)	
IgH translocations					0.645[b]
No	17/64 (26.5)	4/21 (19)	5/14 (35.7)	3/8 (37.5)	
Yes	47/64 (73.4)	17/21 (81)	9/14 (64.3)	5/8 (62.5)	

*$p < 0.05$, **$p < 0.01$. [a], One-way ANOVA followed by LSD test between groups, [b], Two-sided Pearson Chi-Square test.

Supplementary Table 4. Causes of Death in MM (n=326)

Cause of death	No. (%)
Bleeding	23/326 (7)
Infections	35/326 (10.8)
Organ failures	37/326 (11.3)
Progression	191/326 (58.6)
Other Causes	40/326 (12.3)

Supplementary Table 5: related to methods. Summary for primer sequences used in the vector construction

Gene	Direction	Primers sequences
SVIL-ORF	Forward	GACTAGTATGAAAAGAAAAGAAAGAATTGCC
	Reverse	GCTAGCCTTATCGTCGTCATCCTTGTAATCGAA CAGGCCTTTTGCTTTCTTC
SLC38A1-ShRNA-1	Forward	CCGGCCTCCTATTGATCTGTTCAAACTCGAGTT TGAACAGATCAATAGGAGGTTTTTG
SLC38A1-ShRNA-2	Forward	CCGGGCCAATTTACAGTGAGCTTAACTCGAGTT AAGCTCACTGTAAATTGGCTTTTTG
SHMT2-ShRNA	Forward	CCGGCCGAATCAACTTTGCCGTGTTCTCGAGAA CACGGCAAAGTTGATTCGGTTTTTG
MAT2A-ShRNA	Forward	CCGGGCTGTTAAACACATTGGATATCTCGAGATA TCCAATGTGTTTAACAGCTTTTTG
SVIL-ShRNA-1	Forward	CCGGGAGTTCCTCGACCTTCTCTTTCTCGAGAAA GAGAAGGTCGAGGAACTCTTTTTG
SVIL-ShRNA-2	Forward	CCGGCATAAGGAATCTAAATATGCTCTCGAGAGC ATATTTAGATTCCTTATGTTTTTG
SHANK3-shRNA	Forward	CCGGATGCAGTCAGCGGCTGTGGCACTCGAGTG CCACAGCCGCTGACTGCATTTTTG
SETDB2-shRNA	Forward	CCGGCCACATTGGATAATCAGAATACTCGAGTATT CTGATTATCCAATGTGGTTTTTG

Supplementary Table 6: related to methods. Summary for primer sequences used in RT-qPCR assay

**Gene**	**Forward**	**Reverse**
Human–*β-actin*	GTCTTCCCCTCCATCGTG	TTCTCCATGTCGTCCCAG
Human-*SLC38A1*	ATGACAGTGCCCGAGGATGA	TCCAAATGGCTGTTTGTGAGAC
Human-*MAT2A*	GACAGCTCAACGGCTTCCAC	GCATCAAGGACAGCATCACTG
Human-*SVIL*	CAAGAGGTTGCTTTTCAGGGA	AACACGAAGCGGGATAGGT
Human-*SHANK3*	TAAAGGTGCTGAAGAATGGTGGT	TCCTTGTAGTCAGGTGAAGCCC
Human-*HBP1*	TGCCATCTTCACCTGGATATAACT	CTGTTAGCCAGTCCACCTCATTT
Human-*FRYL*	GCAGCTGTACTAGGACTGGGAA	CACTTAGAAATGGTTGTTGAGGC
Human-*ABI2*	GAAGAAACCAAAGCCTACACCAC	TGTGTCCTTGAAGTGTTTTTATTGG
Human-*FAM193A*	AAGTGACGTGGGAACTGCATAATA	TGTCCTCACTGCAGGTGTCGT
Human-*WDTC1*	ATGTGTATTTTGTGGACCTGGG	ACACATCCTGAGTGACCCTGC
Human-*PHF3*	GTTCTGTTTGCCTGTTTTGGATAG	GGCTGTTGTGAGGATCAAGTGAG
Human-*C3oRF52*	TGTGGTTGGAAGATGCAAACTG	GCAACACACTCCTCTGGAATCTT
Human-*MTHFD1*	TTGTTGGCCCAGAAGGGTTT	CTGACAGTGGCAACAAGCAC
Human-*MELK*	AATTACATGAAACTATTGGGACAGG	ACTCCCTAGTGTGTTTTTATCCATG
Human-*STRN3*	TACAAGGCGAAAGAAAAGGTCA	CCTCTGTGTCTTTGGTTTCTTCTG
Human-*SEMA4B*	AGCCTTCAGCCCCATGTGTA	GTTGAGGGAGCTCTCGGTCTT
Human-*MAX*	CGTAGGAAATGAGCGATAACGAT	CGATGAAGGACAGGAGTACACAAT
Mouse-*Phgdh*	AGATTCCCCAGGCAACAGC	CTCTTCCAATTCTGCCGAGG
Mouse-*Psph*	TAGCTGAGCACCCGCCAC	AGCCACCAGAGATGAGGAACA
Mouse–*β-actin*	GGAGGGGGTTGAGGTGTT	GTGTGCACTTTTATTGGTCTCAA

Supplementary Table 7: related to methods. Summary for primer sequences used in ChIP-qPCR and MS-PCR assay

**Gene**	**Direction**	**Primers sequences**	**Application**
GAPDH ChIP	Forward	TACTAGCGGTTTTACGGGCG	ChIP-qPCR
	Reverse	TCGAACAGGAGGAGCAGAGAGCGA	
SVIL ChIP#1	Forward	GAGTAGGGGATAGGGGAGGCT	ChIP-qPCR
	Reverse	GGAGGCTGAGGCAAGAGAATG	
SVIL ChIP#2	Forward	ACCTCGACCGGCTAATTTTTT	ChIP-qPCR
	Reverse	ATAAAAATTGGACTTCAGGGCC	
SVIL ChIP#3	Forward	GGCCCTGAAGTCCAATTTTTAT	ChIP-qPCR
	Reverse	GTCTCCAATTGCATGCCTTTT	
FRYL ChIP#1	Forward	TCTCTTTTCAATGCGTGAGGTTC	ChIP-qPCR
	Reverse	CACCCGGAAGAGCTTTAATCAA	
FRYL ChIP#2	Forward	GATAACTTGATTAAAGCTCTTCCGG	ChIP-qPCR
	Reverse	CCATTATTACTCTCTCTTTACGGGC	
FRYL ChIP#3	Forward	GACTAGCTCTTTCCTTGCCCAA	ChIP-qPCR
	Reverse	GTGGTTAAACCTCCCTCCGA	
SHANK3 ChIP#1	Forward	CAGGCACTGTGCAGAACCC	ChIP-qPCR
	Reverse	GTCTCCCTGCAAGCCTGC	
SHANK3 ChIP#2	Forward	AGGCTTGCAGGGAGACACC	ChIP-qPCR
	Reverse	CCTCGTGTCTCCACCGTCTT	
SHANK3 ChIP#3	Forward	GAGAGACATGGGCAGGAAGAC	ChIP-qPCR
	Reverse	GGAGTCCTAGGCCTCGGTG	
SVIL M	Forward	TATTTTTAGTAGAGACGGGGTTTCGT	MS-PCR
	Reverse	ATACGAAAATTAAACCGAACGC	
SVIL U	Forward	TATTTTTAGTAGAGATGGGGTTTTGTT	MS-PCR
	Reverse	CTAAAAATACAAAAATTAAACCAAACACT	
FRYL M	Forward	TTTTTTTATTAATAAAAATAAAGTTCGT	MS-PCR
	Reverse	CTACAACAAAAACAACAATCCTCG	
FRYL U	Forward	TTTTTTATTAATAAAAATAAAGTTTGT	MS-PCR
	Reverse	CTACAACAAAAACAACAATCCTCAC	
SHANK3 M	Forward	AGAACGTAGGACGTGATTTTTTC	MS-PCR
	Reverse	AAAAAAATCCTAAACCTCGATACG	
SHANK3 U	Forward	GGAGAATGTAGGATGTGATTTTTTT	MS-PCR
	Reverse	AAAAATCCTAAACCTCAATACAAA	
