# Supplementary material for: Excessive serine from the bone marrow microenvironment impairs megakaryopoiesis and thrombopoiesis in Multiple Myeloma
Source: Nat Commun. 2023 Apr 13;14:2093. doi: 10.1038/s41467-023-37699-z (PMC10102122; doi:10.1038/s41467-023-37699-z)
Supplement: Supplementary file 3 — Reporting Summary [file 41467_2023_37699_MOESM3_ESM.pdf]

nature portfolio

Corresponding author(s): Wen Zhou

Last updated by author(s): Mar 9, 2023

Reporting Summary

Nature Portfolio wishes to improve the reproducibility of the work that we publish. This form provides structure for consistency and transparency in reporting. For further information on Nature Portfolio policies, see our Editorial Policies and the Editorial Policy Checklist.

Statistics

For all statistical analyses, confirm that the following items are present in the figure legend, table legend, main text, or Methods section.

n/a	Confirmed	
☐	☒	The exact sample size (*n*) for each experimental group/condition, given as a discrete number and unit of measurement
☐	☒	A statement on whether measurements were taken from distinct samples or whether the same sample was measured repeatedly
☐	☒	The statistical test(s) used AND whether they are one- or two-sided<br>*Only common tests should be described solely by name; describe more complex techniques in the Methods section.*
☐	☒	A description of all covariates tested
☐	☒	A description of any assumptions or corrections, such as tests of normality and adjustment for multiple comparisons
☐	☒	A full description of the statistical parameters including central tendency (e.g. means) or other basic estimates (e.g. regression coefficient) AND variation (e.g. standard deviation) or associated estimates of uncertainty (e.g. confidence intervals)
☐	☒	For null hypothesis testing, the test statistic (e.g. *F*, *t*, *r*) with confidence intervals, effect sizes, degrees of freedom and *P* value noted<br>*Give P values as exact values whenever suitable.*
☒	☐	For Bayesian analysis, information on the choice of priors and Markov chain Monte Carlo settings
☒	☐	For hierarchical and complex designs, identification of the appropriate level for tests and full reporting of outcomes
☐	☒	Estimates of effect sizes (e.g. Cohen's *d*, Pearson's *r*), indicating how they were calculated

Our web collection on statistics for biologists contains articles on many of the points above.

Software and code

Policy information about availability of computer code

Data collection	1. Auto Hematology Analyzer software (mindray), used for complete blood counts.<br>2. SoftMax® Pro 7 software, version 7.1.0 (MolecularDevices), used for ELISA and absorbance.<br>3. Bruker software, version 4.9 (PerkinElmer), used for whole-animal live imaging.<br>4. ChromaTOF software, version 4.71, used for untargeted metabolomics study.<br>5. Masslynx software, version 4.1, used for metabolic flux.<br>6. CFX Manager TM software, version 3.1 (Bio-Rad), used for qRT-PCR.<br>7. LabSolutions CS software (SHIMADZU), used for HPLC.<br>8. CytExpert software (BECKMAN COULTER), or BD FACSDIVA software (BD FACS Canto II or LSRII), used for FACS.
Data analysis	1. GraphPad Prism version 8 was used for data analysis.<br>2. SIMCA14.1 software package was used for orthogonal partial least-squares-discriminant analysis (OPLS-DA) in untargeted metabolomics study.<br>3. IBM SPSS Statistics software, version 18.0 was used for clinical correlation analysis.<br>4. Metabolites in untargeted metabolomics study were annotated with the mammalian metabolite database JiaLibTM using a strict-matching algorithm incorporated in XploreMET software.<br>5. The data of metabolic flux were analyzed by using Targetlynx software, version 4.1 software.<br>6. ImageJ software, version 1.8.0 was used for IF.<br>7. IGV software, version 2.11.9 was used for ATAC-seq analysis.

For manuscripts utilizing custom algorithms or software that are central to the research but not yet described in published literature, software must be made available to editors and reviewers. We strongly encourage code deposition in a community repository (e.g. GitHub). See the Nature Portfolio guidelines for submitting code & software for further information.

Data

Policy information about availability of data

All manuscripts must include a data availability statement. This statement should provide the following information, where applicable:

- Accession codes, unique identifiers, or web links for publicly available datasets
- A description of any restrictions on data availability
- For clinical datasets or third party data, please ensure that the statement adheres to our policy

The raw data of RNA-sequencing and ATAC-seq generated in this study have been deposited in the public database of Genome Sequence Archive (GSA) under the accession number HRA003851and HRA003852, which are accessible at https://bigd.big.ac.cn/gsa-human/browse/HRA003851 and https://bigd.big.ac.cn/gsa-human/browse/HRA003852, respectively. The untargeted metabolomics data and targeted metabolomics data were included in an Excel form which is named as untargeted metabolomic and targeted metabolomic data in the Source Data file. Source data are provided with this paper as a Source Data file. The remaining data are available within the Article, Supplementary Information or Source Data file.

Human research participants

Policy information about studies involving human research participants and Sex and Gender in Research.

Reporting on sex and gender	In the all multiple myeloma patients or clinical samples used for the analysis, 60.5% of the patients were males, 39.5% females.
Population characteristics	All participants are from China. The median age of multiple myeloma patients was 59 with a range spanning between 18-83 years.
Recruitment	The patients and healthy donors were randomly recruited at Xiangya Hospital, the Second, the Third Xiangya Hospital of Central South University, the Blood Diseases Hospital of Chinese Academy of Medical Science & Peking Union Medical College and Peking Union Medical College Hospital of Chinese Academy of Medical Sciences and Peking Union Medical College.
Ethics oversight	Ethics approval was granted by the Ethics Committees of Central South University, Chinese Academy of Medical Sciences and Peking Union Medical College.

Note that full information on the approval of the study protocol must also be provided in the manuscript.

Field-specific reporting

Please select the one below that is the best fit for your research. If you are not sure, read the appropriate sections before making your selection.

☒ Life sciences ☐ Behavioural & social sciences ☐ Ecological, evolutionary & environmental sciences

For a reference copy of the document with all sections, see nature.com/documents/nr-reporting-summary-flat.pdf

Life sciences study design

All studies must disclose on these points even when the disclosure is negative.

Sample size	No Sample size calculation was performed in this study. Sample size was determined according to previous publications. Reference: Jian X, et al. Alterations of gut microbiome accelerate multiple myeloma progression by increasing the relative abundances of nitrogen-recycling bacteria. Microbiome. 8(1):74 (2020).
Data exclusions	No data were excluded from the analyses.
Replication	All in vitro experiments were performed at least three times with similar results. Data described in this manuscript were reliably reproduced.
Randomization	Mice were randomly allocated to control or experimental groups.
Blinding	Investigators were not blinded to sample allocation during experiment and outcome assessment. However, the experimenter were blinded during data collection and analyses.

Reporting for specific materials, systems and methods

We require information from authors about some types of materials, experimental systems and methods used in many studies. Here, indicate whether each material, system or method listed is relevant to your study. If you are not sure if a list item applies to your research, read the appropriate section before selecting a response.

Materials & experimental systems

n/a	Involved in the study
☐	☒ Antibodies
☐	☒ Eukaryotic cell lines
☒	☐ Palaeontology and archaeology
☐	☒ Animals and other organisms
☒	☐ Clinical data
☒	☐ Dual use research of concern

Methods

n/a	Involved in the study
☒	☐ ChIP-seq
☐	☒ Flow cytometry
☒	☐ MRI-based neuroimaging

Antibodies

Antibodies used

1. Bcl-xL Rabbit Polyclonal Antibody, Cat#A0209, Western Blotting 1:1000, ABclonal.
2. SETDB2 Rabbit Polyclonal Antibody, Cat#A7391, Western Blotting 1:1000, ABclonal.
3. SLC1A4 Rabbit Polyclonal Antibody, Cat#A12507, Western Blotting 1:1000, ABclonal.
4. SLC1A5 Rabbit Polyclonal Antibody, Cat#A6981, Western Blotting 1:1000, ABclonal.
5. SHMT2 Rabbit Polyclonal Antibody, Cat#A1215, Western Blotting 1:1000, ABclonal.
6. p53 Rabbit Polyclonal Antibody, Cat#10442-1-AP, Western Blotting 1:1000, Proteintech Group.
7. BAD Rabbit Polyclonal Antibody, Cat#10435-1-AP, Western Blotting 1:1000, Proteintech Group.
8. SLC38A1 Rabbit Polyclonal Antibody, Cat#12039-1-AP, Western Blotting 1:1000, Proteintech Group.
9. Histone-H3 Rabbit Polyconal antibody, Cat#17168-1-AP, Western Blotting 1:5000, Proteintech Group.
10. Beta Actin Mouse Monoconal antibody, Cat#66009-1-Ig, Clone 2D4H5, Western Blotting 1:5000, Proteintech Group.
11. Supervillin Mouse Monoconal antibody, Cat#Sc-53556, Clone B8C1, Western Blotting 1:500, Immunofluorescence, 1:50, Santa Cruz.
12. Tri-Methyl-Histone H3 (Lys9) (D4W1U) Rabbit Monoconal antibody, Cat#13969, Western Blotting 1:1000, Cell Signaling Technology.
13.Cleaved Caspase-3 (Asp175) Rabbit Polyclonal Antibody, Cat#9661S, Western Blotting 1:1000, Cell Signaling Technology.
14. Lineage Cell Detection Cocktail-Biotin, mouse, Cat#130-092-613, Flow cytometry, Miltenyi.
15. CD34 MicroBead Kit, human, Cat#130-046-702, CD34 cell separation, Miltenyi.
16. FITC Rat Anti-Mouse CD41, Cat#561849, Flow cytometry, BD Biosciences.
17. FITC Rat Anti-Mouse CD34, Cat#560238, Flow cytometry, BD Biosciences.
18. FITC Mouse Anti-Human CD41a, Cat#555466, Flow cytometry, BD Biosciences.
19. APC Mouse Anti-Human CD41a, Cat#561852, Flow cytometry, BD Biosciences.
20. PE Mouse Anti-Human CD42b, Cat#561854, Flow cytometry, BD Biosciences.
21. PE Rat Anti-Mouse CD45R/B220, Cat#553089, Flow cytometry, BD Biosciences.
22. APC anti-mouse CD117 (c-Kit) Antibody, Cat#105812, Flow cytometry, Biolegend.
23. PE anti-mouse CD117 (c-Kit) Antibody, Cat#105808, Flow cytometry, Biolegend.
24. FITC anti-mouse CD117 (c-Kit) Antibody, Cat#161604, Flow cytometry, Biolegend.
25. APC/Cyanine7 anti-mouse Ly-6A/E (Sca-1) Antibody, Cat#108126, Flow cytometry, Biolegend.
26. PE anti-mouse CD16/32 Antibody, Cat#101308, Flow cytometry, Biolegend.
27. APC anti-mouse TER-119/Erythroid Cells Antibody, Cat#116212, Flow cytometry, Biolegend.
28. PerCP/Cyanine5.5 anti-mouse CD71 Antibody, Cat#113816, Flow cytometry, Biolegend.
29. PerCP/Cyanine5.5 anti-mouse/human CD45R/B220 Antibody, Cat#103236, Flow cytometry, Biolegend.
30. PerCP/Cyanine5.5 anti-mouse CD105 Antibody, Cat#120416, Flow cytometry, Biolegend.
31. FITC anti-mouse CD3 Antibody, Cat#100204, Flow cytometry, Biolegend.
32. PE anti-mouse CD138 (Syndecan-1) Antibody, Cat#142504, Flow cytometry, Biolegend.
33. PE/CY7-conjugated streptavidin,Cat#405206, Flow cytometry, Biolegend.

Validation

All antibodies for western blotting and flow cytometry are commercially available and were validated by the manufacturer as follows:
1. Bcl-xL Rabbit Polyclonal Antibody, Cat#A0209, ABclonal. The antibody was validated by Western Blotting, IHC and IF. The figures of Western Blotting, Immunohistochemistry and Immunofluorescence are listed on the manufacturer's website. (https://abclonal.com.cn/catalog/A0209).
2. SETDB2 Rabbit Polyclonal Antibody, Cat#A7391, ABclonal. The antibody was validated by Western Blotting and IF. The figures of Western Blotting are listed on the manufacturer's website. (https://abclonal.com.cn/catalog/A7391).
3. SLC1A4 Rabbit Polyclonal Antibody, Cat#A12507, ABclonal. The antibody was validated by Western Blotting. The figures of Western Blotting are listed on the manufacturer's website. (https://abclonal.com.cn/catalog/A12507).
4. SLC1A5 Rabbit Polyclonal Antibody, Cat#A6981, ABclonal. The antibody was validated by Western Blotting. The figures of Western Blotting are listed on the manufacturer's website. (https://abclonal.com.cn/catalog/A6981).
5. SHMT2 Rabsbit Polyclonal Antibody, Cat#A1215, ABclonal.T he antibody was validated by Western Blotting, IHC, IF and IP. The figures of Western Blotting are listed on the manufacturer's website. (https://abclonal.com.cn/catalog/A1215).
6. P53 Rabbit Polyclonal Antibody, Cat#10442-1-AP, Proteintech Group. The manufacturer states that this antibody can be used for Western Blotting, IF, IP, ELISA. The antibody was used in 580 publications. The antibody was validated by Western Blotting in 4 cell lines, including A549, HepG2, MCF7, and SMMC-7721. The figure of Western Blotting is listed on the manufacturer's website. (https://www.ptgcn.com/products/P53-Antibody-10442-1-AP.htm).
7. BAD Rabbit Polyclonal Antibody, Cat#10435-1-AP, Proteintech Group. The manufacturer states that this antibody can be used for Western Blotting, FC, IHC and ELISA. The figures of Western Blotting, IHC and FC are listed on the manufacturer's website. (https://www.ptgcn.com/products/BAD-Antibody-10435-1-AP.htm).
8. SLC38A1 Rabbit Polyclonal Antibody, Cat#12039-1-AP, Proteintech Group. The manufacturer states that this antibody can be used for Western Blotting, IP, IHC, ELISA. The antibody was used in 7 publications (https://www.ptgcn.com/products/SLC38A1-Antibody-12039-1-AP.htm).
9. Histone-H3 Polyclonal antibody, Cat#17168-1-AP, Proteintech Group. The manufacturer states that this antibody can be used for

Western Blotting, IP, IHC, ELISA. The antibody was used in 457 publications (https://www.ptgcn.com/products/Histone-H3-Antibody-17168-1-AP.htm).
10. Beta Actin Mouse Monoconal antibody, Cat#66009-1-Ig, Proteintech Group. The manufacturer states that this antibody can be used for Western Blotting, IP, IHC, IF, FC, CoIP, ChIP, ELISA. The antibody was used in 2789 publications (https://www.ptglab.com/products/Pan-Actin-Antibody-66009-1-Ig.htm).
11. Supervillin Mouse Monoconal antibody, Cat#Sc-53556, Santa Cruz.he manufacturer states that this antibody can be used for Western Blotting, IP, IF. The antibody was used in 1 publications (https://www.scbt.com/p/supervillin-antibody-b8c1).
12. Tri-Methyl-Histone H3 (Lys9) (D4W1U) Rabbit Monoconal antibody, Cat#13969, Cell Signaling Technology. The manufacturer states that this antibody can be used for Western Blotting, IP, IF, FC, ChIP. The antibody was used in 72 publications (https://www.cellsignal.cn/products/primary-antibodies/tri-methyl-histone-h3-lys9-d4w1u-rabbit-mab/13969?site-search-type=Products&N=4294956287&Ntt=h3k9me3&fromPage=plp).
13.Cleaved Caspase-3 (Asp175) Rabbit Polyclonal Antibody, Cat#9661S, Cell Signaling Technology. The manufacturer states that this antibody can be used for Western Blotting, IF, IP, IHC, FC. The antibody was used in 7055 publications (https://www.cellsignal.com/products/primary-antibodies/cleaved-caspase-3-asp175-antibody/9661?site-search-type=Products&N=4294956287&Ntt=cleaved+capase-3+%28asp175%29+&fromPage=plp).
14. Lineage Cell Detection Cocktail-Biotin, mouse, Cat#130-092-613, Miltenyi. The manufacturer states that this antibody can be used for Flow cytometry. (https://www.miltenyibiotec.com/CN-en/products/lineage-cell-detection-cocktail-biotin-mouse.html#130-092-613).
15. CD34 MicroBead Kit, human, Cat#130-046-702, Miltenyi. The manufacturer states that this antibody can be used for the positive selection of CD34+ hematopoietic stem and progenitor cells in a single step. (https://www.miltenyibiotec.com/CN-en/products/cd34-microbead-kit-human.html#130-046-702).
16. FITC Rat Anti-Mouse CD41, Cat#561849, BD Biosciences. The manufacturer states that this antibody can be used for Flow cytometry. (https://www.bdbiosciences.com/zh-cn/products/reagents/flow-cytometry-reagents/research-reagents/single-color-antibodies-ruo/fitc-rat-anti-mouse-cd41.561849).
17. FITC Rat Anti-Mouse CD34, Cat#560238, BD Biosciences. The manufacturer states that this antibody can be used for Flow cytometry. (https://www.bdbiosciences.com/zh-cn/products/reagents/flow-cytometry-reagents/research-reagents/single-color-antibodies-ruo/fitc-rat-anti-mouse-cd34.560238).
18. FITC Mouse Anti-Human CD41a, Cat#555466, BD Biosciences. The manufacturer states that this antibody can be used for Flow cytometry. (https://www.bdbiosciences.com/zh-cn/products/reagents/flow-cytometry-reagents/research-reagents/single-color-antibodies-ruo/fitc-mouse-anti-human-cd41a.555466).
19. APC Mouse Anti-Human CD41a, Cat#561852, BD Biosciences. The manufacturer states that this antibody can be used for Flow cytometry. (https://www.bdbiosciences.com/zh-cn/products/reagents/flow-cytometry-reagents/research-reagents/single-color-antibodies-ruo/apc-mouse-anti-human-cd41a.561852).
20. PE Mouse Anti-Human CD42b, Cat#561854, BD Biosciences. The manufacturer states that this antibody can be used for Flow cytometry. (https://www.bdbiosciences.com/zh-cn/products/reagents/flow-cytometry-reagents/research-reagents/single-color-antibodies-ruo/pe-mouse-anti-human-cd42b.561854).
21. PE Rat Anti-Mouse CD45R/B220, Cat#553089, BD Biosciences. The manufacturer states that this antibody can be used for Flow cytometry. (https://www.bdbiosciences.com/zh-cn/products/reagents/flow-cytometry-reagents/research-reagents/single-color-antibodies-ruo/pe-rat-anti-mouse-cd45r-b220.553089).
22. APC anti-mouse CD117 (c-Kit) Antibody, Cat#105812, Biolegend. The manufacturer states that this antibody can be used for Flow cytometry. The antibody was used in 65 publications (https://www.biolegend.com/en-us/products/apc-anti-mouse-cd117-c-kit-antibody-72)
23. PE anti-mouse CD117 (c-Kit) Antibody, Cat#105808, Biolegend. The manufacturer states that this antibody can be used for Flow cytometry. The antibody was used in 31 publications (https://www.biolegend.com/en-us/products/pe-anti-mouse-cd117-c-kit-antibody-75)
24. FITC anti-mouse CD117 (c-Kit) Antibody, Cat#161604, Biolegend. The manufacturer states that this antibody can be used for Flow cytometry. (https://www.biolegend.com/en-us/products/fitc-anti-mouse-cd117-c-kit-antibody-20973)
25. APC/Cyanine7 anti-mouse Ly-6A/E (Sca-1) Antibody, Cat#108126, Biolegend. The manufacturer states that this antibody can be used for Flow cytometry. The antibody was used in 27 publications (https://www.biolegend.com/en-us/products/apc-cyanine7-anti-mouse-ly-6a-e-sca-1-antibody-6752)
26. PE anti-mouse CD16/32 Antibody, Cat#101308, Biolegend. The manufacturer states that this antibody can be used for Flow cytometry. The antibody was used in 27 publications (https://www.biolegend.com/en-us/products/pe-anti-mouse-cd16-32-antibody-189)
27. APC anti-mouse TER-119/Erythroid Cells Antibody, Cat#116212, Biolegend. The manufacturer states that this antibody can be used for Flow cytometry. The antibody was used in 52 publications (https://www.biolegend.com/en-us/products/apc-anti-mouse-ter-119-erythroid-cells-antibody-1863)
28. PerCP/Cyanine5.5 anti-mouse CD71 Antibody, Cat#113816, Biolegend. The manufacturer states that this antibody can be used for Flow cytometry. The antibody was used in 2 publications (https://www.biolegend.com/en-us/products/percp-cyanine5-5-anti-mouse-cd71-antibody-11662)
29. PerCP/Cyanine5.5 anti-mouse/human CD45R/B220 Antibody, Cat#103236, Biolegend. The manufacturer states that this antibody can be used for Flow cytometry. The antibody was used in 58 publications (https://www.biolegend.com/en-us/products/percp-cyanine5-5-anti-mouse-human-cd45r-b220-antibody-4267)
30. PerCP/Cyanine5.5 anti-mouse CD105 Antibody, Cat#120416, Biolegend. The manufacturer states that this antibody can be used for Flow cytometry. The antibody was used in 4 publications (https://www.biolegend.com/en-us/products/percp-cyanine5-5-anti-mouse-cd105-antibody-9225)
31. FITC anti-mouse CD3 Antibody, Cat#100204, Biolegend. The manufacturer states that this antibody can be used for Flow cytometry. The antibody was used in 101 publications (https://www.biolegend.com/en-us/products/fitc-anti-mouse-cd3-antibody-45)
32. PE anti-mouse CD138 (Syndecan-1) Antibody, Cat#142504, Biolegend. The manufacturer states that this antibody can be used for Flow cytometry. The antibody was used in 31 publications (https://www.biolegend.com/en-us/products/pe-anti-mouse-cd138-syndecan-1-antibody-7519)
33. PE/CY7-conjugated streptavidin,Cat#405206, Flow cytometry, Biolegend.The manufacturer states that this antibody is useful for detecting biotinylated antibodies. The antibody was used in 46 publications (hhttps://www.biolegend.com/en-us/products/pe-cyanine7-streptavidin-1477)

Eukaryotic cell lines

Policy information about cell lines and Sex and Gender in Research

Cell line source(s)	ARP1 and RPMI-8226 cell lines were obtained from Cancer Research Institute, Central South University. OPM2 cell line was obtained from the Institute of Hematology and Blood Diseases Hospital, Chinese Academy of Medical Sciences. Luciferase-labeled 5TGM1 cell line was kindly provided by Prof. Yuhuan Zheng (West China Center of Medical Sciences, Sichuan, China).
Authentication	All multiple myeloma cell lines were authenticated by short tandem repeat (STR) DNA profiling analysis.
Mycoplasma contamination	Cell lines used in this study are negative for mycoplasma.
Commonly misidentified lines (See ICLAC register)	No commonly misidentified cell lines used in this study.

Animals and other research organisms

Policy information about studies involving animals; ARRIVE guidelines recommended for reporting animal research, and Sex and Gender in Research

Laboratory animals	6-8-week-old male or female C57BL/KaLwRij mice were used in this study. All mice were maintained under SPF conditions in a controlled environment of 20–22 °C, with a 12/12 h light/dark cycle, 50–70% humidity.
Wild animals	No wild animals used in this study.
Reporting on sex	Both male and female mice were applied for in vivo tumor progression.
Field-collected samples	No field-collected samples were used.
Ethics oversight	Animals experiments in this study were compiled with ethical regulations and approved by the Ethics Committees of Central South University and Hunan Normal University (animal experimental license number: D2021016).

Note that full information on the approval of the study protocol must also be provided in the manuscript.

Flow Cytometry

Plots

Confirm that:

☒ The axis labels state the marker and fluorochrome used (e.g. CD4-FITC).

☒ The axis scales are clearly visible. Include numbers along axes only for bottom left plot of group (a 'group' is an analysis of identical markers).

☒ All plots are contour plots with outliers or pseudocolor plots.

☒ A numerical value for number of cells or percentage (with statistics) is provided.

Methodology

Sample preparation	The Flow Cytometry Analysis was descriptived in "Methods" section
Instrument	CytoFLEX S Flow Cytometer (Beckman Coulter), BD FACS CantoII or BD LSRII Flow cytometer (BD Biosciences).
Software	FlowJo v10.0.7 (BD)
Cell population abundance	The purity of interesting cell population higher than 95% was used for further experiment.
Gating strategy	To identify the cells population of interesting, the forward vs side scatter (FSC-A versus SSC-A) was used. The FSC-A/FSC-H were used to remove the doublets from total cell population. Interesting cells were gated out based on gating of isotype control and single stained cells using appropriate channels.

☒ Tick this box to confirm that a figure exemplifying the gating strategy is provided in the Supplementary Information.
