# Supplementary material for: Excessive serine from the bone marrow microenvironment impairs megakaryopoiesis and thrombopoiesis in Multiple Myeloma
Source: Nat Commun. 2023 Apr 13;14:2093. doi: 10.1038/s41467-023-37699-z (PMC10102122; doi:10.1038/s41467-023-37699-z)
Supplement: Supplementary file 4 — Description of Additional Supplementary Files [file 41467_2023_37699_MOESM4_ESM.docx]

**Description of Additional Supplementary Files**

File Name: Supplementary Data 1

Description: related to Figure 4.

Differential genes highly correlated with serine in cells undergoing MK differentiation by RNA-seq

File Name: Supplementary Data 2

Description: related to Figure 5.

metabolic flux of serine in cells undergoing MK differentiation

File Name: Supplementary Data 3

Description: related to Figure 5.

Differential peak related genes in cells undergoing MK differentiation at day 12 by ATAC-seq.
